# Modified Robert Jones bandage can not reduce postoperative swelling in enhanced-recovery after primary total knee arthroplasty without intraoperative tourniquet: a randomized controlled trial

**DOI:** 10.1186/s12891-018-2281-6

**Published:** 2018-10-05

**Authors:** Haoda Yu, Haoyang Wang, Kai Zhou, Xiao Rong, Shunyu Yao, Fuxing Pei, Zongke Zhou

**Affiliations:** Department of Orthopedics, West China Hospital, Sichuan University, Chengdu, 610041 China

**Keywords:** Total knee arthroplasty, Swelling, Modified Robert jones bandage, Compression

## Abstract

**Background:**

Compression therapy is commonly used to reduce lower limb swelling and blood loss after knee surgery. This study was performed to investigate whether modified Robert Jones bandage (MRJB) as a postoperative compression therapy is necessary for enhanced-recovery primary total knee arthroplasty without the tourniquet application.

**Methods:**

In this prospective randomized controlled trial, 90 patients were grouped into 2 groups randomly. The experimental group received compression therapy with MRJB from toes to thigh for 24 h and the control group received no compression therapy. Knee swelling, blood loss, range of motion (ROM), pain, patient reported comfort level and complications were recorded.

**Results:**

No significant differences were observed between the two groups when we compared knee swelling. Similarly, no significant difference on postoperative blood loss, pain, ROM, complications was found. However, patients in control group had significantly higher comfort ratings than compression group during the first 24 h.

**Conclusions:**

MRJB is not routinely indicated in enhanced-recovery primary total knee arthroplasty without tourniquet application.

**Trial registration:**

The trial was registered in the Chinese Clinical Trial Registry (ChiCTR-INR-16010177) dated 18th December 2016.

## Background

Total knee arthroplasty (TKA) is a common and highly successful orthopedic operation to relieve pain and improve knee function in people with end-stage knee osteoarthritis [1]. However, TKA is associated with the high prevalence of postoperative knee swelling, which results in decreased knee-extension strength and impaired functional performance [2]. Knee swelling is due to intra-articular bleeding and peri-articular inflammation. Postoperative compression therapy with Modified Robert Jones bandage (MRJB) from toes to mid-thigh is commonly performed in patients who underwent arthroplasty with the hypothesis that it could reduce intra-articular bleeding by providing tamponade effect in the knee and reduce soft tissue edema by increasing intra-tissular pressure, aiding venous return in the lower limb [3, 4]. However, if the bandage is too tight, the excessive external pressure could lead to tissue ischemia by obliterating blood flow to subcutaneous tissue [5]. Compression-related complications including bruises, blisters, peroneal nerve palsy and discomforts complaint from patients have been reported by various researchers [6–8].

Recently, the growing trend of quicker recovery following orthopedic procedures has stimulated the development of the techniques focused on reducing post-operative knee swelling. In addition to compression, various postoperative methods including surgery without intraoperative tourniquet use, administration of tranexamic acid (TXA) and corticosteroid medication have been reported to be effective to reduce hemarthrosis and soft tissue edema [9, 10], as this multi-modal swelling management could effectively reduce post-operative hidden blood loss and limit inflammation. However, few studies have adequately investigated and demonstrated the benefits of MRJB when applied together with this multi-modal swelling management, resulting in a knowledge gap and inability to determine if MRJB is still necessary for patients undergoing primary TKA in an enhanced recovery after surgery (ERAS) program. Therefore, we conducted this prospective randomized controlled trial (RCT) to evaluate the effect of using MRJB on knee swelling, blood loss, pain, complications and patient-reported knee function after TKA. We hypothesized that MRJB is not necessary when this multi-modal swelling management is utilized in enhanced-recovery after primary total knee arthroplasty.

## Methods

### Study design

This prospective, randomized controlled study was approved by the institutional review board of West China Hospital of Sichuan University (no. 201302009) and registered in the Chinese Clinical Trial Registry (ChiCTR-INR-16010177). All patients, aged 18 years or older, who were scheduled for a primary total knee arthroplasty for end-stage osteoarthritis were eligible for inclusion. Exclusion criteria included simultaneously bilateral total knee arthroplasty or revision case, surgical history of the knee joint, peripheral vascular disease, ankle brachial pressure index, ABPI< 0.8, peripheral neuropathy, blood coagulation disorders, history of deep venous thrombosis, BMI > 35, knee stiffness characterized as flexion deformity of ≥30°. Informed consent was obtained from all participants.

### Treatment groups

**Fig. 1:**
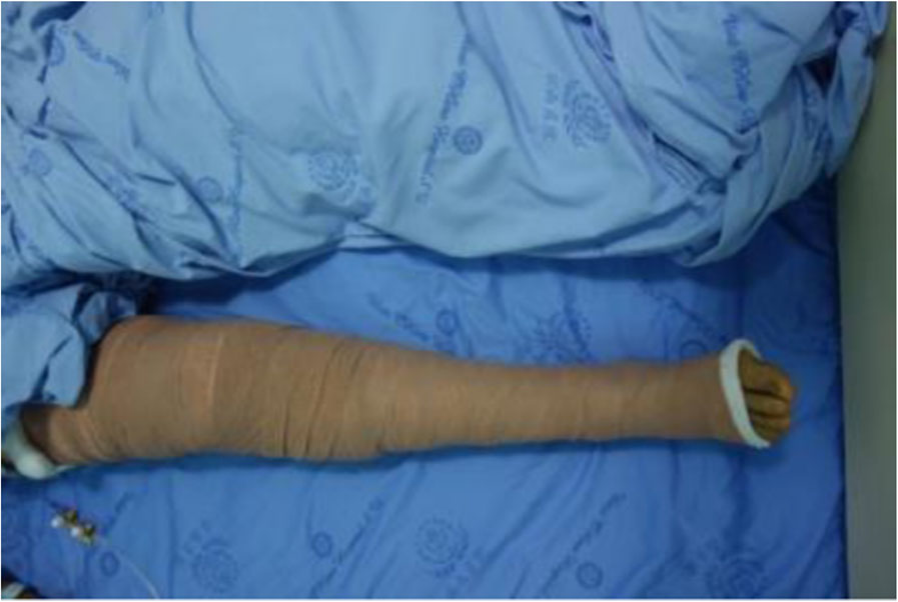
A MRJB is shown on a patient

**Fig. 2:**
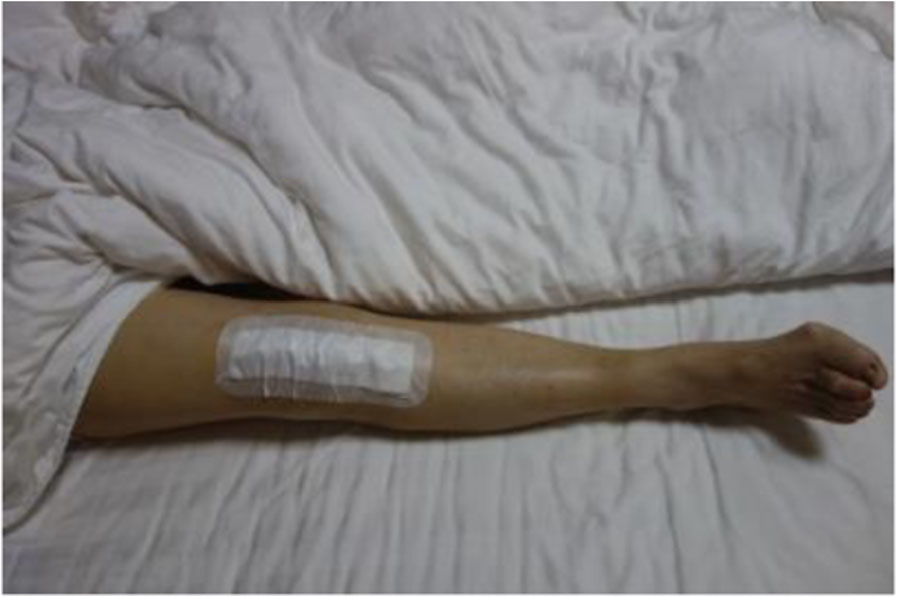
A conventional wound dressing is shown on a patient

Recruited patients were randomly allocated to either the MRJB or the conventional wound dressing group according to a computerized random sequence generator. After wound closure, sequentially numbered, sealed envelopes were opened in the operating room. In the MRJB group, the sterile adhesive wound dressing Cosmopor®E (Paul Hartmann AG, Heidenheim, Germany) was placed over the wound followed by a soft inner layer thick cotton padding Winner (Chengdu Wenjian Likang Medical Products Ltd., Sichuan, China) which was applied from toes to thigh. The outer layer was composed of elastic bandage Coban™ (3 M Deutschland GmbH, Neuss, Germany). To enhance venous return, more tension was applied distally than proximally (Fig. 1). The MRJB retained for 24 h postoperatively, while the conventional wound dressing group was treated with sterile adhesive wound dressing over the wound only (Fig. 2). The surgeons were blinded to the treatment assignment until wound closure, and data collector were blinded during the entire study.

### Surgical procedures

All TKAs were performed by the same surgical team by using a midline skin incision, a standard medial parapatellar approach, and a measured resection technique. A cemented posterior-stabilized prosthetic total knee prosthesis (PFC, Johnson & Johnson/DePuy, Warsaw, IN, USA) was used. All of the patients received an intravenous administration of TXA 5 to 10 min before the skin incision (20 mg/kg) and 3, 6 h later (1 g) along with 1 g of topical TXA in 50 mL of normal saline solution. No tourniquet or wound drainage was used in any patient and no blood salvage system was used. Electrocautery and routine hemostasis were performed during the surgery. Surgical time, intraoperative blood loss volume was recorded. All patients had adductor canal block (20 ml 5 g/L ropivacaine and 0.1 mg adrenaline) performed before surgery and periarticular infiltration analgesia (70 ml 2.5 g/L ropivacaine and 0.1 mg adrenaline) during surgery.

### Postoperative care protocol

After the operation, the patients were transferred to the anesthesia recovery unit, where they remained for 1 h, and then to the bed-ward. A cold pack was used on the surgical site and a single dose of 10 mg intravenous dexamethasone was administered when patients arrived inpatient unit [9, 11]. Postoperative oral Loxoprofen sodium (Loxonin; 60 mg three times daily) and Pregabalin (Lyrica; 75 mg twice daily) were administered for pain [12, 13]. A standard venous thromboembolism prophylaxis protocol combined mechanical and chemical prophylaxis was adopted for all patients [9]. An intermittent inflatable lower-extremity pump was used as a routine practice to prevent deep venous thrombosis (DVT) before the patient began walking. Low-molecular-weight heparin (LMWH; Clexane, Sanofi-Aventis, France, 2000 IU) was administered subcutaneously 8 h postoperatively, and a full dose (0.4 mL containing 4000 IU) was given at 24-h intervals during hospitalization. After discharge, rivaroxaban (10 mg, Xarelto, Bayer, Germany) was administered orally for 10 days if no bleeding events occurred.

Criteria for transfusion included a Hb level less than 7 g/dL or symptomatic anemia (light-headedness, palpitation, or shortness of breath not due to other causes) in a patient with an Hb level of 7 to 10 g/dL [9].

### Outcome measurements

**Fig. 3:**
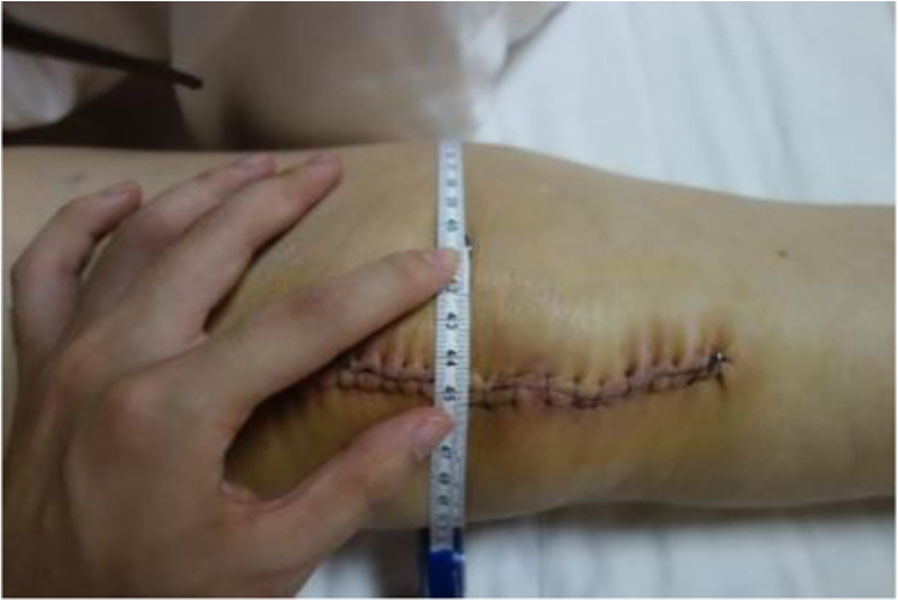
Measurement of swelling

The primary outcome was swelling, which was measured as circumference of superior pole of patella, inferior pole of patella, mid-line of patella, thigh (10 cm above superior pole of the patella) and calf (10 cm below to inferior pole of the patella) at postoperative day (POD) 1, POD3 and 3 weeks after surgery. The secondary outcomes included range of motion (ROM), hospital for special surgery knee score, visual analog scale (VAS) at rest and at walking, reduction in Hb concentration, postoperative calculated blood loss, complications and patient satisfaction. The blood volume of each patient was calculated according to a formula published by Nadler et al. that considers patients’ weight, height and gender [14, 15]. Complications such as nerve palsy, bruises, blisters were recorded. Doppler ultrasound was used to evaluate for DVT when a patient has any suspicious symptom of DVT, including severe pain, swelling, tenderness, superficial venous engorgement and Homan’s sign. Pulmonary embolism (PE) was diagnosed by clinical symptoms and an enhanced chest computed tomography (CT) scan. All adverse events were recorded during the first 3 weeks after surgery (Fig. 3). All of the patients completed a comfort level questionnaire regarding the feeling of the operated lower limb that asked them to rate their comfort on a 5-point scale ranging from very uncomfortable to very comfortable.

### Statistical analysis

Quantitative data were presented as mean and standard deviation, qualitative data were presented as size number. Differences in continuous variables between the two groups were evaluated using Student’s t-test or Mann–Whitney U test, depending on the distribution characteristics of the data. A chi-square test or Fisher’s exact test for difference in proportions was used to estimate differences between groups in categorical variables. The sample size estimate was based on the difference in the primary outcome (i.e., reduction in mean knee circumference) and calculation was performed using G*Power Version 3.1.9.2 (Franz Faul, Uni Kiel, Germany) software with an unpaired t test of variance design assuming a standard effect size (d) = 0.67, an alpha level (two-tailed) = 0.05, and power = 0.90. We took 2 cm as clinical minimal relevance in circumference based on a previous study [16], and assumed standard deviation within each group to be 3 cm. Based on the information mentioned above, 39 patients each arm were needed. Allowing for a 15% loss to follow up, a total of 90 patients were planned to include in this study.

## Results

### Patients’ demographics

During recruitment from December 2016 to May 2018, a total of 453 patients scheduled to take a primary unilateral TKA were screened. Of them all, 143 patients were ineligible, 220 declined to participate, and the remaining 90 participants were enrolled. Each group lost 1 patient during follow-up. Baseline demographic data were comparable between the 2 groups (Table 1).

**Table 1:** Demographic characteristic of patients

	Compression group	Control group	*P* value
Age (yr)	69.32 ± 8.29	69.11 ± 8.66	0.91
^a^Gender (Male/Female)	10/34	10/34	1
Height (m)	1.58 ± 0.09	1.59 ± 0.08	0.64
Weight (kg)	64.39 ± 9.47	64.67 ± 10.82	0.90
BMI (kg/m^2^)	25.80 ± 2.88	25.60 ± 3.24	0.76
Circumference (cm)			
Superior pole of patella	39.53 ± 2.91	39.47 ± 3.64	0.93
Mid-line of patella	38.31 ± 2.71	38.13 ± 3.28	0.78
Inferior pole of patella	36.31 ± 2.85	36.18 ± 3.15	0.83
Thigh	44.83 ± 36.59	44.64 ± 4.30	0.82
Calf	34.07 ± 2.43	33.79 ± 2.52	0.60
Hemoglobin (g/dL)	132.23 ± 12.55	134.09 ± 12.84	0.49
Hematocrit (%)	40.25 ± 3.25	40.11 ± 3.38	0.85
ROM	96.55 ± 14.22	92.91 ± 14.04	0.23
Pain at walking(VAS)	6.23 ± 1.27	6.34 ± 1.20	0.59
Pain at rest(VAS)	1.39 ± 1.71	1.14 ± 1.49	0.55

BMI body mass index, ROM range of motion, VAS visual analogue scale

^a^Presented as number and percent, and *P*-values were calculated by chi-square and Fisher exact test

### Primary outcome

No significant differences were observed between the two groups when we compared knee circumference at superior margin of patella, inferior margin of patella, mid-line of patella at any measurement point (Table 2).

**Table 2:** Postoperative primary outcomes

Variable	Compression group	Control group	*P* value
Mean circumference (cm)			
Superior pole of patella			
POD1	41.75 ± 2.90	41.61 ± 3.46	0.84
POD3	42.64 ± 2.88	42.56 ± 3.50	0.91
3w	40.19 ± 2.89	40.11 ± 3.57	0.91
POD1 change	2.22 ± 1.01	2.14 ± 0.86	0.71
POD3 change	3.10 ± 1.31	3.08 ± 1.12	0.95
3w change	0.66 ± 0.26	0.64 ± 0.25	0.80
Mid-line of patella			
POD1	40.40 ± 2.66	40.26 ± 3.28	0.84
POD3	41.31 ± 2.62	41.03 ± 3.37	0.67
3w	38.97 ± 2.62	38.82 ± 3.25	0.82
POD1 change	2.09 ± 0.96	2.13 ± 0.93	0.81
POD3 change	3.00 ± 1.32	2.90 ± 1.27	0.73
3w change	0.66 ± 0.31	0.69 ± 0.26	0.56
Inferior pole of patella			
POD1	38.08 ± 2.71	37.97 ± 3.22	0.87
POD3	38.88 ± 3.11	38.53 ± 3.54	0.63
3w	36.92 ± 2.81	36.73 ± 3.17	0.77
POD1 change	1.76 ± 1.14	1.79 ± 0.98	0.90
POD3 change	2.57 ± 1.63	2.35 ± 1.46	0.52
3w change	0.61 ± 0.36	0.56 ± 0.31	0.48
Thigh			
POD1	46.23 ± 3.49	45.90 ± 4.19	0.68
POD3	46.87 ± 3.68	46.57 ± 4.22	0.72
3w	45.79 ± 3.46	45.53 ± 4.14	0.74
POD1 change	1.40 ± 0.71	1.26 ± 0.66	0.35
POD3 change	2.03 ± 0.96	1.93 ± 0.90	0.61
3w change	0.96 ± 0.61	0.89 ± 0.58	0.58
Calf			
POD1	35.20 ± 2.44	34.95 ± 2.63	0.65
POD3	35.30 ± 2.52	35.07 ± 2.67	0.68
3w	34.82 ± 2.39	34.54 ± 2.46	0.60
POD1 change	1.12 ± 0.66	1.16 ± 0.62	0.83
POD3 change	1.22 ± 0.78	1.27 ± 0.70	0.75
3w change	0.74 ± 0.56	0.75 ± 0.43	0.95

*POD* postoperative day, 3w 3 weeks after surgery

### Secondary outcome

No significant differences were observed in the total blood loss between the 2 groups. Similarly, transfusion rate showed no significant difference. As to the postoperative general assessments, no significant difference on Pain, ROM, HSS was found (Table 3). However, patients in control group had significantly higher comfort ratings than the experimental group during the first 24 h (Table 4). No nerve palsy, PE or DVT was observed in the 2 groups. Superficial skin complications including bruises and blisters also showed no significant difference.

**Table 3:** Postoperative secondary outcomes

Variable	Compression group	Control group	*P* value
Postoperative Hb drop (g/dL)			
POD1 drop	18.91 ± 7.68	18.45 ± 8.17	0.79
POD3 drop	28.75 ± 10.01	29.27 ± 12.21	0.83
Postoperative blood loss (mL)			
POD1 drop	252.48 ± 124.92	250.75 ± 106.60	0.94
POD3 drop	529.30 ± 232.62	558.94 ± 252.74	0.57
Intraoperative blood loss (mL)	188.70 ± 80.33	178.14 ± 77.65	0.53
VAS at walking			
*POD1	6.23 ± 1.89	5.89 ± 1.62	0.37
*POD3	2.16 ± 0.94	1.98 ± 0.93	0.46
VAS at rest			
*POD1	2.39 ± 1.91	1.91 ± 1.60	0.28
*POD3	0.39 ± 0.72	0.41 ± 0.62	0.59
ROM (°)			
POD1	77.50 ± 20.00	78.30 ± 17.39	0.84
POD3	99.16 ± 9.36	97.68 ± 10.43	0.49
HSS	80.55 ± 5.36	79.34 ± 4.66	0.26
Complications			
^†^DVT	0	0	–
^†^PE	0	0	–
^†^Transfusion	0	0	–
^†^Bruises	1	1	1
^†^Blisters	1	1	1
^†^Nerve palsy	0	0	–

*POD* postoperative day, *VAS* visual analogue scale, *ROM* range of motion, *HSS* hospital for special surgery knee score, *DVT* deep vein thrombosis, *PE* pulmonary embolism

*P values calculated using the Mann–Whitney U test

^†^presented as number and the *P*-values were calculated by chi-square and Fisher exact test

**Table 4:** Comfort level at different time points

Comfort level	Compression group	Control group	*P* value
*Postoperative 24 h			0.03
Very comfortable	0	0	
Somewhat comfortable	2	4	
Fair	6	12	
Somewhat uncomfortable	27	24	
Very uncomfortable	9	4	
*Postoperative 72 h			0.58
Very comfortable	1	1	
Somewhat comfortable	7	8	
Fair	20	21	
Somewhat uncomfortable	13	13	
Very uncomfortable	3	1	

*P values calculated using the Mann–Whitney U test

## Discussion

Hidden blood loss and inflammation are believed to be main factors that caused post-operative swelling. Based on this mechanism, various methods have been introduced to reduce post-operative knee swelling. Tourniquet-induced ischemia could increase fibrinolytic activity and induce local reactive hyperemia, resulting in more hidden blood loss [17]. Thus, abandoning tourniquet could lead to less hidden blood loss and a lower ratio of postoperative knee swelling [9]. Ishida et al. reported that the intra-articular administration of TXA reduced knee swelling by diminishing the hidden blood loss [18]. Furthermore, both TXA and corticosteroid have anti-inflammatory effects which could also contribute to reduction of swelling [19, 20]. All of the methods mentioned above are now routinely used in our institution for enhanced recovery.

Modified Robert Jones bandage, which was introduced by Brodell, is one of the most common compressive dressing following orthopedics surgery during the last 30 years, with the benefits of reducing tissue bleeding and edema by increasing intramuscular and intraarticular pressures [21]. However, Concerns about the complications such as peroneal nerve palsy, bruises and patient-reported discomforts still remain [6–8]. Due to the effective modern multi-modal swelling management, modified Robert Jones bandage use after total knee arthroplasty may now be unnecessary. To our best knowledge, the present study was the first to evaluate the effect of MRJB in an ERAS program after TKA without use of a tourniquet and post-operative drains.

In this study, we found significantly lower patient comfort level in the compression group during the first 24 h, but no significant difference between the 2 groups at POD3, when MRJB had been removed for patients in the compression group. This was in accord with the previous study, compression therapy was with poor patients’ compliance [4]. One of the reasons was reported to be the compression induced discomfort factors, including obliteration of circulation, “too hot” to wear, limb soreness, dermatitis or itching [22]. Moreover, low-pressure compression therapy was determined to be more comfortable than the high-pressure compression therapy [23].

Another finding of our study was that there was no difference in swelling, ROM and total blood loss between the 2 groups, and this was similar with a RCT performed by Pinsornsak et al. In their study, MRJB were placed for 24 h after TKA in the compression group, while patients in the control group received no compression but conventional wound dressing with sterile gauze pads only [6]. The difference between their study and ours was that they performed with routine tourniquet, no administration of intra-articular TXA or post-operative corticoids. In contrast, Charalambides et al. reported that compression bandage could control intra-articular bleeding effectively and few patients with compression bandaging experienced post-operative lower limb swelling [3]. However, the study was not in an enhanced recovery setup and the swelling was not evaluated by knee circumference. One of the possible reasons for the positive result of Charalambides et al.’s study may be the bandages were maintained for 48 h, which was at twice the time of ours. And this may be too long for patients to achieve early mobilization and may be conflict to the idea of ERAS, as this bandage is bulky and hard for patients to do flexion exercise.

Furthermore, we found no differences in pain relief between the two groups. However, in a study evaluating the effect of compression bandage on pain control, Andersen et al. found patients with compression bandage experienced less pain than those with non-compression bandage [24]. This might be due to the cooling effect of cryotherapy in our study was partly affected by MRJB because of its thick layer [25], as cryotherapy was reported to reduce pain by slowing the conduction of nerve signals [26].

There are several limitations in our study. First, we did not measure the inflammatory markers before surgery, so the level of inflammation could be confounding issue and may have effect on knee swelling. However, the effect may be negligible due to the randomization design. Second, sub-bandage pressure measurement was not performed in each patient with the MRJB dressing, so the interface pressures might differ with each application. However, measurement of sub-bandage pressure is not practical and a pressure-guided application method of MRJB was not routinely used in clinical practice. Third, patients were not blinded in this study, as it was difficult to prevent patients from noticing if they received compression or not. Fourth, during our clinical practice, we found intraoperative tourniquet was still necessary for patients with severe obesity because of their greater surgical difficulty, so we excluded patients with a BMI > 35. Therefore, our conclusion may not be applicable for patients with severe obesity.

## Conclusions

In conclusion, we found avoidance of MRJB use could provide higher patients’ reported comfort level, without increasing swelling, blood loss, severity of pain or damaging knee function. Therefore, MRJB after primary TKA without tourniquet and drainage may not be routinely indicated in common clinical use.

## Abbreviations

CT: Computed tomography
DVT: Deep venous thrombosis
MRJB: Modified Robert Jones bandage
PE: Pulmonary embolism
POD: Postoperative day
ROM: Range of motion
TKA: Total knee arthroplasty
VAS: Visual analog scale
